# Acceptability of Antenatal Multiple Micronutrient Supplementation (MMS) Compared to Iron and Folic Acid (IFA) Supplementation in Pregnant Individuals: A Narrative Review

**DOI:** 10.3390/nu17182994

**Published:** 2025-09-18

**Authors:** Mihaela C. Kissell, Carolina Pereira, Filomena Gomes, Kidist Woldesenbet, Masresha Tessema, Hiwot Kelemu, Ramadhani Noor, Luz Escubil, Aishwarya Panicker, Ashutosh Mishra, Mai-Anh Hoang, Hou Kroeun, Cassandra Sauer, Meng Sokchea, Crystal D. Karakochuk, Masako Horino, Keith P. West, Akihiro Seita, Djeinam Toure, Umu H. Jalloh, Francis Moses, Aminata S. Koroma, Bakary Diarra, Ousmane Camara, Ouassa Sanogo, Kristine Garn, Martin N. Mwangi

**Affiliations:** 1 Healthy Mothers Healthy Babies Consortium, Micronutrient Forum, Washington, DC 20005-3915, USA; 2 NOVA Medical School, Universidade NOVA de Lisboa, 1169-056 Lisboa, Portugal; 3 Federal Ministry of Health, Addis Ababa P.O. Box 1234, Ethiopia; 4 Ethiopia Public Health Institute, Addis Ababa P.O. Box 1242, Ethiopia; 5 UNICEF Ethiopia, Addis Ababa P.O. Box 1169, Ethiopia; 6 The Vitamin Angel Alliance, Goleta, CA 93117, USA; 7 Helen Keller Intl, No. 42 St 322, Phnom Penh 12302, Cambodia; 8 Human Nutrition, The University of British Columbia, Vancouver, BC V6T 1Z4, Canada; 9 BC Children’s Hospital and Individuals’ Health Research Institutes, Vancouver, BC V5Z 4H4, Canada; 10 United Nations Relief and Works Agency for Palestine Refugees in the Near East (UNRWA), Amman 11814, Jordan; 11 Center for Human Nutrition, Department of International Health, Johns Hopkins Bloomberg School of Public Health, Baltimore, MD 21205, USA; 12 Helen Keller Intl, Africa Region Office, Dakar 29898, Senegal; 13 Helen Keller Intl, Freetown, Sierra Leone; 14 Reproductive Health and Family Planning, Ministry of Health and Sanitation, Freetown, Sierra Leone; 15 Food and Nutrition, Ministry of Health and Sanitation, Freetown, Sierra Leone; 16 Institut National de Santé Publique, Bamako 1771, Mali; 17 Helen Keller Intl, Bamako 1557, Mali; 18 Division of Human Nutrition and Health, Wageningen University & Research (Bode 62), P.O. Box 17, 6700 AA Wageningen, The Netherlands

**Keywords:** acceptability, adherence, antenatal care, iron and folic acid (IFA), low- and middle-income countries (LMICs), maternal nutrition, multiple micronutrient supplements (MMS), pregnancy

## Abstract

**Background/Objectives**: Antenatal multiple micronutrient supplementation (MMS) improves birth outcomes more effectively than iron and folic acid (IFA) supplementation alone. However, the acceptability of MMS among pregnant individuals, a critical factor for adherence and program success, remains poorly defined and inconsistently assessed. This narrative review proposes a comprehensive definition of “acceptability” in the context of nutritional supplementation and evaluates the evidence on the acceptability of MMS compared to IFA in low- and middle-income countries (LMICs). **Methods**: We conducted a systematic literature search across Embase, Medline, and Scopus to identify studies (including grey literature) reporting on acceptability-related outcomes for MMS versus IFA among pregnant individuals. Studies exploring dimensions such as organoleptic properties, ease of consumption, side effects, cultural appropriateness, and socioeconomic factors were included. **Results**: Out of 1056 screened studies, five informed a novel multi-dimensional definition of acceptability. Six studies assessed acceptability-related characteristics. MMS was generally accepted across most organoleptic domains. Most studies reported fewer or comparable adverse side effects for MMS as compared to IFA. Studies consistently reported more perceived benefits for MMS than IFA. Facilitating factors included trust in health professionals, free provision, and family support. Barriers included poor taste or smell, fear of side effects, misconceptions, cost, and lack of family support. **Conclusions**: Antenatal MMS is widely acceptable in LMICs. Addressing socio-cultural, sensory, and socioeconomic factors is essential to increase uptake and adherence. This review provides a clear, standardized definition of acceptability to guide future research and inform effective program design.

## 1. Introduction

Micronutrient deficiencies remain a major public health concern worldwide, particularly among individuals of reproductive age. Recent global estimates have shown that more than half of the global population consumes inadequate levels of several micronutrients essential to health, including iron, calcium, and vitamins C and E [1]. Globally, two out of three individuals aged 15–49 years are deficient in iron, zinc, or folate [2]. Anemia alone affects ~37% (or 34 million) of pregnant individuals globally, posing risks for maternal morbidity, fetal development, and neonatal survival [3]. Pregnant individuals have a higher risk of anemia due to physiological changes that the mother and the developing fetus experience [4].

Since 1968, the World Health Organization (WHO) has recommended daily iron and folic acid (IFA) supplementation during pregnancy as part of routine antenatal care (ANC) to prevent and treat maternal anemia [5,6]. However, growing evidence from clinical trials and meta-analyses over the past two decades shows that MMS, particularly the United Nations International Multiple Micronutrient Antenatal Preparation (UNIMMAP) formulation containing 15 essential vitamins and minerals, offers additional benefits beyond IFA alone [7]. MMS is considered safe and efficacious in reducing the risk of low birth weight, preterm birth, being small for gestational age, and stillbirths [8,9], as well as reducing the chances of giving birth to small vulnerable newborns with the greatest mortality risk [10], while promoting better infant growth up to 6 months of age [11]. Among anemic pregnant women, the magnitude of the benefits of MMS are even greater (as compared to non-anemic pregnant women), with a demonstrated 19% risk reduction of low birthweight and a 29% risk reduction of 6-month infant mortality [9]. While there is interim guidance encouraging the concurrent use of MMS during anemic treatment in pregnant women [12], future research is needed to determine the ideal dose of additional iron when the likely cause of anemia is iron deficiency [13].

WHO’s 2020 global antenatal care guidelines conditionally recommended MMS in the context of “rigorous research,” recognizing the need to assess the impact of transitioning from IFA to MMS, including evaluation of the acceptability, feasibility, sustainability, equity, and cost-effectiveness of MMS compared to IFA [14]. Among these, acceptability of a healthcare intervention is broadly defined as participants’ perception that the intervention is appropriate and their willingness to receive the intervention as intended, while considering anticipated or experienced cognitive and emotional responses of the intervention [15]. This plays a pivotal role in adherence and program impact [16,17]. Yet, despite growing interest in MMS, the concept of acceptability has received limited and inconsistent attention in the nutrition literature. Often conflated with adherence, “acceptability” lacks a standardized definition and validated measurement tools in the nutrition field, making it difficult to compare findings across studies or inform large-scale implementation.

Definitions for acceptability are more commonly used in the healthcare field [15] and specifically in pharmacology, as proposed by Marant [18] and Weiner [19]. Overall, these authors point out that acceptability helps understand a patient’s adherence to a medicine. Although each expert came up with a separate definition, they all emphasize that the intervention should be centered around the patient’s anticipated and experienced responses of the intervention.

The acceptability of nutritional supplements has not been uniformly studied in the nutrition literature, with authors measuring and applying different characteristics of acceptability, such as organoleptic properties, the benefits of intervention, convenience, etc. [20,21,22,23,24]. To address this critical knowledge gap, the current narrative review proposes a standardized and comprehensive definition of acceptability in the context of antenatal nutritional supplementation for future use in nutrition research and programming. It also evaluates evidence from the published and grey literature on the acceptability of MMS compared to IFA among pregnant individuals in LMICs. By doing so, the review aims to inform the design of more responsive and culturally appropriate supplementation programs.

## 2. Materials and Methods

The primary aim of this narrative review was to propose a comprehensive definition of “acceptability” as applicable in the nutrition field. The proposed definition was applied to study the acceptability of MMS compared to IFA supplementation during pregnancy. To achieve this, we conducted a literature search of published research articles and the grey literature available from inception until 5 January 2024, and updated it on 27 May 2025, in three databases: Embase, Medline, and Scopus. We also reached out to experts conducting research on MMS acceptability to identify unpublished case studies. The search strategy was developed without year restrictions but was limited to English language publications using the following keywords: “micronutrients,” “dietary supplements,” “supplementation,” “pregnancy,” “adherence,” “compliance,” and “acceptability” (see Table S1 for the detailed strategy used in Ovid Medline). Of note, the keyword “adherence” was included, as some people use acceptability and adherence interchangeably. The inclusion and exclusion criteria applied to titles and abstracts are shown in Table 1.

## 3. Results

The systematic literature search conducted in January 2024 and updated in May 2025 retrieved 1056 results. After applying the inclusion criteria (Table 1), 491 duplicates were removed, and out of the remaining 565, 24 articles were selected for full text review, of which nine were included in the present study (see Supplemental Figure S1). Of these, five articles focused on the domains of acceptability of MMS versus IFA (or MMS only) in individuals of reproductive age (primarily pregnant individuals) and informed the new definition of “acceptability.” Six articles assessed various dimensions of acceptability in studies providing MMS versus IFA to pregnant individuals. We extracted information on the following factors: country of study, intervention type (groups and form), dose of iron (mg), adherence rates (because acceptability informs adherence), composition of supplements, physical appearance, utilization patterns, adverse side effects, organoleptic properties (size, taste/flavor, color, texture, and smell/odor), ease of use and convenience, perceived benefits or effectiveness, negative perceptions, delivery methods, and female autonomy. Separately, we obtained three case studies (unpublished) that focused on MMS acceptability in pregnant women from LMICs (see Supplementary Materials, Case Studies S1–S3).

### 3.1. Acceptability Definition and Key Concepts

As previously mentioned, we identified five studies [20,21,22,23,24] that tested the acceptability of MMS (three out of five compared MMS with IFA, with the rest examining MMS only) in individuals of reproductive age. However, not all authors specified the definition of acceptability that they chose to follow in their studies; instead, they collected information on different characteristics of acceptability. Once we extracted and analyzed the information relevant to acceptability from each study (as detailed in Table 2), we proposed the following standardized definition for future use: “Acceptability is the (comprehensive assessment of) a pregnant individual’s willingness and satisfaction in integrating the intervention (i.e., MMS) into their daily routine and involves evaluating factors such as sensory attributes (e.g., taste), ease of consumption, and overall patient experience (e.g., adverse side effects), recognizing cultural nuances and individual preferences. It extends beyond adherence, encompassing cultural appropriateness, socio-economic considerations, and the overall compatibility of the MMS with individual preferences and lifestyles.” This definition was reviewed and approved by the Global MMS Technical Advisory Group [25].

### 3.2. Acceptability of MMS Versus IFA in Pregnancy Studies

Separately, we extracted information from six studies (five peer-reviewed and one conference abstract) that focused on MMS (in comparison with IFA) interventions given during pregnancy and reported on acceptability constructs (Table 3, Table 4 and Table 5). Of these six studies, four were conducted in Africa (one in Niger [20], two in Mali [22,26], and one in Ethiopia [27]), one in the Middle East (in Jordan, called UNRWA (United Nations Relief and Works Agency for Palestine Refugees in the Near East Health Systems [28]), and one in Southeast Asia (in Cambodia [29]).

As shown in Table 3, there is variability in the study designs. The dose of iron differed in MMS and/or IFA across most studies, such that MMS contained 30 mg and IFA most often contained 60 mg (Table 3).

For the majority of studies, the MMS and IFA came in the form of tablets (Table 4), and three out of the six studies provided a UNIMMAP MMS formulation (Table 3). The appearance of the tablets did not differ between intervention groups in the Aguayo study in Mali [22], but in all other studies, either the form of the tablet or the packaging differed (Table 4).

Regarding utilization of the intervention, there was variability in the instructions given for the interventions. In the Clermont study in Niger [20], individuals in the MMS group were given four tablets/day and were told to take two tablets in the morning and two tablets in the evening with water after a meal. Individuals in the IFA group were given one tablet/day, to be taken with water after a meal in the evening. These instructions, along with guidelines for storage, were first given by the study midwife at enrolment and then by health assistants during weekly home visits [20]. Although precise instructions were given, they were followed more closely in the IFA than the MMS groups; for instance, many individuals in the MMS group opened the gel capsule and mixed the contents with food. In the Aguayo study, individuals were told to take one tablet/day with water before going to bed (see precise guidelines in Table 4). These instructions were provided at the beginning of the second trimester until delivery and until 3 months postpartum (90 days of tablets every 3 months; [22]). In the Sauer study, Cambodian individuals were told of the benefits of supplementation and that they should take one tablet every night before going to bed [29]. Other studies did not report on this construct (Table 4).

Adverse side effects were similar across MMS and IFA groups in two studies [20,22], but interestingly, in the Mali study by Aguayo that provided supplementation for the first 3 months postpartum, the authors observed no adverse side effects as reported in either group during the postpartum period [22]. In all other studies that compared the two supplements, there were more adverse side effects reported in the IFA than in the MMS group. Of note, the Cambodian study tracked adverse side effects over time and found higher reporting at 30 vs. 90 days in both the MMS and IFA groups, with adverse effects higher in the IFA group at both time points. By 180 days, side effects were even lower, though data at this point were available only for the MMS groups [29]. Next, in an MMS study in Mali [26], individuals who took IFA in a prior pregnancy and now were taking MMS reported less nausea and vomiting with MMS. Also, some individuals who were taking IFA during pregnancy reported feeling unwell and nauseous; when they were switched to MMS, they reported no more nausea. The most reported adverse side effects were vomiting, nausea, and dizziness (Table 4).

Organoleptic properties such as size, taste/flavor, color, and smell were rated similarly in the Aguayo study [22]. Most individuals reported that the taste of MMS was better than IFA in the studies by Horino and Sauer. For size, Aguayo found no difference between MMS and IFA, but Sauer and Clermont found that individuals liked the size of MMS more than of IFA. For color, it either did not differ between groups [22] or individuals preferred the color of MMS to that of IFA [29] or shared that they would prefer the MMS pill color to be red rather than white or green ([27]; Table 4). With respect to smell, one study in Mali found no difference [22] between groups. More Cambodian pregnant individuals reported they liked the MMS smell vs. IFA [29], whereas Horino reported the opposite in pregnant refugee individuals [28]. Lastly, other constructs rarely measured in studies include texture, ease of use, or convenience. Next, for ease of use or consumption, in the Aguayo study in Mali, there was no difference between intervention groups. In the Clermont study in Niger, some individuals reported that if they had no food, they would skip the supplement, and in the Ethiopian study that only assessed IFA utilization, individuals reported avoiding taking IFA if they had no food, due to gastrointestinal discomfort (Table 4; [27]).

Most studies consistently reported higher perceived benefits in the MMS rather than IFA group [20,26,28,29,30]. For example, in the Clermont study, which asked individuals about specific health claims, both MMS and IFA groups indicated increased appetite and improved health of mother and baby; however, more individuals in the IFA than MMS group reported increased blood volume (Table 5; [20]). In the Aguayo study, there was no difference between groups for these characteristics, and all individuals said that the supplement was beneficial during pregnancy and eliminated pregnancy-related side effects [22]. One study [27] that only assessed this characteristic in individuals receiving MMS reported positive health benefits for mom and baby.

Facilitating factors: the Niger individuals in the Clermont study indicated a high level of trust in doctors, the public health system, free healthcare, and knowing that supplements help with a fast delivery. In the Aguayo, Abebe, and Sauer studies, most individuals indicated that it is easy to remember to take the supplement daily and that they were encouraged by family and community members to take their supplement (also reported in two other studies [26,27]). Other important facilitating factors that were observed consistently across studies were fewer adverse side effects with MMS rather than IFA, MMS being offered free of cost or at a similar cost as IFA, and improved appetite. Based on the Ba study, individuals who took IFA during a prior pregnancy and were now taking MMS indicated that they preferred MMS over IFA.

Common negative perceptions or barriers to consumption included bad taste and smell, fear of adverse side effects, misconceptions (e.g., MMS seen as a drug/medicine, baby will grow too big, painful delivery, and IFA causes hemorrhage during delivery), not taking the supplement if lacking food, forgetfulness, lack of support from husbands and elderly individuals, and having to pay for the supplement (Table 5).

Delivery of the intervention was mainly via ANC health centers, and in two studies, it was delivered by community health workers at home [20,22]. In most populations studied, family members, especially husbands and elderly individuals, had an important role in the decision-making process of individuals as related to pregnancy care. For instance, three studies that assessed female autonomy directly indicated that individuals often relied on husbands or in-laws for approval to attend the ANC or take prenatal supplements [20,22,26].

Although we did not focus this review on intervention adherence, four studies measured it in the context of other acceptability constructs, and the rates of adherence are shown in Table 3. The methods of assessment included self-report with random household spot checks, tablet count, and bottle weight. Overall, despite some individuals reporting negative characteristics in MMS rather than IFA, based on three studies [22,28,29], when comparing MMS with IFA using similar methodologies, we observed consistently that adherence rates were higher in individuals who took MMS rather than IFA (all higher than 80%).

Last, the (unpublished) case studies were conducted in The Philippines (Supplementary Case Study S1), Sierra Leone (Supplementary Case Study S2), and Mali (Supplementary Case Study S3). Two out of three were formative studies, and one had a pre/post design (IFA given first, then MMS); all studies provided a UNIMMAP MMS formulation that contained 30 mg of elemental iron, and for the case of IFA, 60 mg of elemental iron. Regarding the product appearance and utilization, all case studies showed that pregnant individuals liked the package and said it was easy to use, especially when compared to IFA. The most common adverse side effects were nausea and vomiting, and in the Filipino study, 9% stopped taking MMS due to adverse side effects. Next, when organoleptic properties were measured, pregnant individuals liked the size, taste, color, and smell of the MMS tablets, but Malian pregnant individuals reported the tablet smell and size being less acceptable to swallow. Perceived benefits were similar to those from the published studies and included health benefits to mother and baby. The facilitated factors that were unique to the case studies included finding the use of a pill dispenser convenient as it reminded participants to take the supplement. Last, limited female autonomy was also observed in these studies as well as in the published ones.

## 4. Discussion

This narrative review presents a comprehensive framework for assessing the acceptability of nutritional supplements and synthesizes evidence on the acceptability of MMS compared to IFA among pregnant individuals. It responds to a critical gap in maternal nutrition research and programming: the lack of a standardized, multidimensional definition of “acceptability” in the context of antenatal supplementation. Acceptability is central to sustained adherence and, by extension, the effectiveness of maternal nutrition interventions. Yet, it remains inconsistently defined and measured across studies.

Only five studies explicitly assessed acceptability, with varying definitions and inconsistent criteria (not all focused on pregnancy). To address this, we drew from the broader healthcare literature to propose a robust definition encompassing sensory, ease of consumption, adverse side effects, and cultural and socio-economic factors.

Drawing from both the healthcare and nutrition literature, and by examining dimensions such as sensory attributes, cultural relevance, ease of use, and perceived benefits, we propose a broader working definition of “acceptability” as a pregnant woman’s willingness and satisfaction in integrating a supplement into her daily routine. This expanded definition incorporates sensory attributes, ease of consumption, perceived health benefits, and broader cultural and socio-economic dimensions. Unlike adherence, which reflects actual intake, acceptability encompasses the cognitive and emotional responses that influence willingness to initiate and sustained use of the intervention. By clearly distinguishing between these two constructs, this review provides a conceptual foundation for future research and implementation.

This gap is especially relevant, as more than 30 countries [31] are at various stages of transitioning from the provision of IFA to MMS, with Indonesia being the first to nationally transition. In addition, Sierra Leone is the first country to scale up MMS nationwide, focusing on government-owned and government-assisted health facilities. Program implementers are keen to circumvent factors that hinder the success of IFA supplementation, such as associated adverse side effects, poor adherence, and inadequate supply. This underscores the urgency of understanding and addressing enablers and barriers to supplement use from the perspective of end users [32,33].

Our analysis of six relevant studies (five peer-reviewed and one conference abstract) highlights that MMS is more widely accepted than IFA, particularly with respect to organoleptic properties, perceived benefits, and adverse side effect profiles. These findings add to the mounting evidence on the superior health outcomes associated with MMS compared to IFA. Key factors influencing acceptability included supplement composition, palatability, appearance, dosing, instructions for use, adverse side effects, perceived benefits (e.g., improved energy, appetite, and pregnancy outcomes), and family or community support. We discuss the key themes that emerged from the studies included below.

Sensory experiences: Individuals consistently rated MMS more favorably than IFA across sensory domains, particularly in taste, smell, and color. The poor organoleptic properties of IFA (e.g., metallic taste, unpleasant odor, larger size) have long been barriers to adherence. MMS, particularly in the UNIMMAP formulation, often addresses these issues, although challenges remain in some settings. Innovations for improving smell and taste include enteric coating of the pills, but this may increase the cost of the supplement [34].

Perceived health benefits: Perceptions of improved appetite, energy, fetal growth, and maternal well-being were frequently cited as facilitators of MMS use. Importantly, these perceptions are often held regardless of supplement type, underscoring the role of effective health communication in shaping user experience. Studies where individuals transitioned from IFA to MMS noted higher satisfaction and preference for MMS.

Adverse side effects: Across multiple studies, adverse side effects were more common with IFA than MMS, likely due to higher iron content (60 mg in IFA vs. 30 mg in MMS [35]). Some studies suggested that negative side effects may stem from pregnancy itself rather than the supplements [22,29]. Counseling on managing undesirable side effects and simplifying instructions, such as taking the supplements with a meal or before going to bed, can enhance acceptability and, in turn, increase adherence. Since gastrointestinal discomfort is a known deterrent to IFA adherence, this advantage of MMS may support higher long-term adherence. Nevertheless, some reported adverse side effects may reflect pregnancy-related symptoms rather than supplement characteristics, suggesting the need for nuanced counseling strategies.

Sociocultural and systemic barriers: Acceptability is shaped not only by the supplement itself but also by structural and relational dynamics. Common barriers included limited female autonomy, the need for spousal or elder approval, negative community perceptions, misconceptions (e.g., MMS causing larger babies), poor taste, and economic constraints. In several studies, individuals required permission from husbands or elders to attend ANC facilities or take nutritional supplements. However, targeted education in both pregnant individuals and their husbands improved support for MMS use. Community engagement and gender-sensitive programming are thus essential for scaling MMS successfully. Facilitators of acceptability included trust in healthcare providers, supportive counseling, free or affordable access, and family support, highlighting the need to deliver MMS together with counseling by healthcare providers and involving family members as much as possible.

Variability in definitions and tools hinders study comparisons: The diversity of definitions and constructs used to assess “acceptability” in the literature challenges cross-study comparisons. Many studies report on only a subset of relevant characteristics or blend acceptability with adherence. Standardizing definitions and developing validated tools to capture multiple domains, ranging from sensory feedback to cultural conformity, is urgently needed.

Adherence was generally high, especially for MMS rather than IFA (over 80% for studies with similar methods), despite some individuals reporting more negatively for MMS than IFA on organoleptic properties; other factors that might have influence adherence may potentially be due to clear instructions, perceived health benefits, frequent messaging, and minimum adverse side effects. Individuals in MMS groups often reported more positive outcomes than those in IFA groups for the acceptability domains. These cumulative factors suggest that acceptability can improve adherence. Notably, some studies found that perceived benefits—such as healthier babies and improved maternal well-being—were reported regardless of the supplement type, suggesting the importance of health messaging.

This review’s strength lies in its inclusive and comprehensive scope, incorporating diverse published studies from ongoing initiatives in various LMIC settings. This inclusive approach allowed us to capture a wide range of factors influencing MMS and IFA acceptability. However, limitations include the small number of studies assessing acceptability, heterogeneity in study designs, and potential context-specific findings, which may limit the generalizability of findings. The lack of standardized tools for measuring acceptability further complicates comparisons across studies. Furthermore, acceptability measures were often embedded within broader implementation evaluations rather than assessed through standalone methodologies.

Addressing the factors identified in this review is crucial to enhancing the acceptability and, ultimately, the effectiveness of antenatal supplementation programs. Tailored health education, culturally sensitive messaging for supplement use instructions, and ensuring the acceptability, availability, and affordability of MMS are critical components of successful implementation. Future research should focus on developing tools to measure the various dimensions of MMS acceptability.

## 5. Conclusions

In conclusion, as LMICs increasingly consider the adoption of MMS in routine antenatal care, understanding and addressing the acceptability of these supplements becomes crucial. Our review shows that MMS is widely acceptable among pregnant individuals, often preferred over IFA, and associated with fewer adverse side effects and more perceived health benefits. Acceptability is multidimensional, encompassing not only physical and sensory aspects but also cultural, relational, and systemic influences. Our proposed definition offers a starting point for standardizing how acceptability is conceptualized and measured in maternal nutrition programs. Future research should apply the proposed definition, prioritize the development of validated tools for assessing acceptability (distinctly from adherence), and explore how this construct interacts with adherence and health outcomes, while critically assessing data quality. To improve maternal and neonatal outcomes, countries must go beyond efficacy and address the lived experiences of individuals using these supplements. This includes developing context-specific education strategies, strengthening antenatal counseling, involving family and community influencers, and ensuring that MMS is both accessible and affordable. The introduction of MMS presents a unique opportunity to improve the health delivery system, and each country will have to identify its own set of barriers and facilitators influencing the effective delivery of MMS to pregnant women and their adherence to MMS [36].

## Figures and Tables

**Table 1:** Inclusion/exclusion criteria.

Criteria	Inclusion	Exclusion
Population	Pregnant individuals *	Non-pregnant individuals, other non-relevant populations
Intervention	Studies reporting on or comparing the acceptability or adherence of MMS versus IFA	Studies not reporting on the acceptability of MMS or IFA
Outcome of interest	Patient acceptability defined by factors such as composition, palatability (size, shape, texture), appearance (color, shape), required dose (number of tablets per dose), dosing frequency, treatment duration, side effects, and ease of use Adherence (as defined by study authors) Preferences of pregnant individuals regarding MMS over IFA	Studies that do not provide information on patient acceptability or adherence or preferences
Study design	Both quantitative and qualitative studies examining patient acceptability, including randomized controlled trials (RCTs), observational studies, qualitative research, and reports, case studies	Animal studies, and studies with insufficient information on acceptability
Language	English	Studies in languages for which translation resources are not available

* For the definition of acceptability, studies reporting the perspectives of health workers and women of reproductive age were also included for a more comprehensive approach.

**Table 2:** Definitions or constructs of patient acceptability as used in the healthcare and nutrition field, especially when assessing MMS acceptability in pregnant individuals.

First Author	Population (Life Stage)	Definition
Aguayo [22]	Individuals (pregnancy and lactation)	Perceived benefits and/or side effects, perceptions (supplement size, color, taste or flavor, smell), easy to remember taking supplement, ease of use, encouragement vs. discouragement from family or community members, adherence.
Young [21]	Individuals (pregnancy and lactation)	Acceptability looks at sensory characteristics (taste, texture, odor), ease of use (packaging, preparation, portion size, storage, form of consumption), and how side effects, a regular supply or lack thereof, the cultural constructs of medicines, and perceived efficacy have affected use (positive perceived health benefits vs. negative perceived health consequences).
Klevor [24]	Individuals (pregnancy and lactation)	Favorable attitude toward a product, predisposing a person to be willing to use it according to instructions; the study assessed sensory attributes (taste, smell, palatability, texture, color), side effects, food practices, need perceptions and benefits, and social environment.
Clermont [20]	Individuals (pregnancy)	Supplement acceptability, consumption practices, facilitating factors and barriers, perceived side effects, perceived benefits, support or opposition from household members (e.g., husbands and in-laws), and the supplement delivery mechanism.
Silubonde [23]	Individuals (non-pregnancy)	Part 1: Barriers and facilitating factors for MMS adherence for health workers (barriers: knowledge of anemia, lack of experienced benefits, experienced side effects, family support; facilitators: knowledge of anemia, perceived benefits, family support, counseling from community health worker, access to MMS). Part 2: Participant’s understanding and motivation for supplement use, their concerns and emotions around medication, family and peer beliefs around medication, family and peer expectations and need for supplements, and family and peer beliefs around anemia diagnosis.

**Table 3:** Individual study characteristics of MMS vs. IFA (or MMS only) interventions in pregnant individuals.

Study	Study Setting	Study Design	Intervention Groups	Dose of Iron (mg)	Adherence
Clermont [20]; Niger	Rural area in south-central Niger	Randomized trial	MMS vs. IFA vs. LNS	MMS: 30 mg IFA: 60 mg	LNS > IFA > MMS (collected differently than the rest of the studies)
Aguayo [22]; Mali	Two health districts close to Bamako	Effectiveness study	MMS vs. IFA	MMS: 30 mg IFA: 60 mg	95.4% MMS > 92.2% IFA
Ba [26]; Mali	6 health facilities in and around Bamako	Qualitative study	MMS (UNIMMAP; two groups) vs. IFA	MMS: 30 mg IFA: 60 mg	N/A
Sauer [29]; Cambodia	Semi-rural with peri-urban population (largely agrarian economy)	Non-blinded cluster-randomized 3-arm parallel, non-inferiority trial	MMS (two groups) vs. IFA	MMS: 30 mg IFA: 60 mg	95% MMS groups > 91% IFA (adherence did not differ between MMS groups)
Abebe [27]; Ethiopia *	21 districts in 5 regions	Formative study	MMS (UNIMMAP) vs. IFA	MMS: 30 mg IFA: 30–60 mg	N/A
Horino [28]; Jordan (UNRWA) Conference abstract	Palestinian refugees living across Jordan and those in refugee camps (high % of anemia)	Quasi-randomized implementation trial	MMS (UNIMMAP; 180-count bottle) vs. FA/IFA (FA in the 1st trimester and IFA thereafter; 10 blister packs)	MMS: 30 mg IFA: 100 mg	82% MMS > 69% FA/IFA

* The study tested IFA utilization, whereas for MMS, their willingness to pay for MMS was assessed; Abbreviations: FA = Folic Acid Supplementation, IFA = Iron and Folic Acid Supplementation, LNS = Lipid-based Nutrient Supplement, MMS = Multiple Micronutrient Supplementation, N/A = Not Applicable, UNIMMAP MMS = The United Nations International Multiple Micronutrient Antenatal Preparation.

**Table 4:** Acceptability characteristics of MMS vs. IFA or MMS-only interventions in pregnant individuals.

Study	Composition/ Form	Appearance	Utilization	Adverse Side Effects	Size	Taste/Flavor	Color	Smell/Odor
Clermont [20]; Niger	MMS: white powder inside a gel capsule IFA: red tablet	MMS: plastic bottle with 40 gel capsules IFA: blister pack of 10 red tablets	MMS: 4 tablets/day, 2 in the morning and 2 in the evening with water after meal IFA: 1 tablet/day with water after evening meal	↔; vomiting, nausea, dizziness, and weakness	MMS > IFA	Few said IFA tasted bad; some individuals in the MMS group opened the gel capsules and mixed the contents with food	Not measured	Few said IFA smelled bad; when MMS capsule was opened, few individuals reported bad odor
Aguayo [22]; Mali	MMS and IFA: sachet with 90 tablets	MMS and IFA: identical tablets	MMS and IFA: take one tablet (and only one) daily for the benefit of mother health and that of the baby; take the tablet before going to bed to minimize potential undesirable side effects; drink a glass of water to help swallow the tablet; keep the tablet out of reach of children	↔; vomiting, nausea, headache, dizziness	↔; acceptable size	↔	↔	↔; 29/30 said MMS/IFA smelled bad
Ba [26]; Mali	MMS (1): 30-count bottles MMS (2): 180-count bottles IFA: 30-count blister packs	MMS: 30 or 180-tablet bottles IFA: 30-tablet blister packs	Midwives provided counseling during ANC visits, instructions with photos and messages on how to take MMS, and a calendar to track supplementation intake	MMS < IFA; nausea, vomiting, unwell	Not measured	Not measured	Not measured	Some reported IFA had a worse smell compared to MMS
Sauer [29]; Cambodia	MMS (1): 180 tablet bottles MMS (2): 2 × 90 tablet bottles IFA: 90 tablets in a clear plastic bag	MMS > IFA MMS: 180 tablets at ANC1 or 2 × 90 tablets at ANC1 and ANC2 IFA: 90 tablets	Health care workers indicated: “Supplements are good for the health of the mother and baby. They help the baby be strong and smart.” “Take [tablet] every night before going to bed.”	MMS < IFA; stomach cramping, constipation, diarrhea, headache, nausea, heartburn, tiredness, trouble sleeping, or other	MMS > IFA	MMS > IFA	MMS > IFA	MMS > IFA
Abebe [27]; Ethiopia *	N/A	MMS: 38% red, 27% white, 15% green pills	IFA: individuals received instructions and tracking information	IFA: nausea, burning pain, discomfort, constipation	Not measured	IFA: bad taste	MMS: participants preferred red > white > green color tablets	Not measured
Horino [28]; Jordan (UNRWA) Conference abstract	MMS: 180 tablets Folic acid: 10 count blister pack (1st trimester) and IFA: 10 count blister packs thereafter	MMS: 180-tablet bottles IFA: 10-tablet blister packs	MMS clinics: Posters promoting MMS were displayed, women received instructional pamphlets on MMS. In MMS and IFA clinics, women received standard antenatal care education materials.	MMS < IFA; 73% vs. 48% reported no stomach upset/constipation through the first 3 follow-up intervals	Not measured	MMS > IFA	Not measured	MMS < IFA; 28% vs. 46% said tablets smelled good

* The study tested IFA utilization, whereas for MMS their willingness to pay for MMS was assessed; ↔ no difference between groups; Abbreviations: ANC = Antenatal Care, IFA = Iron and Folic Acid Supplementation, MMS = Multiple Micronutrient Supplementation, N/A = Not Applicable.

**Table 5:** Facilitating and barrier factors of MMS versus IFA (or MMS only) in pregnant individuals.

Study	Perceived Benefits/Effectiveness	Facilitating Factors	Negative Perceptions/Barriers to Consumption	Delivery Method of Supplement	Female Autonomy
Clermont [20]; Niger	Increased appetite ↔ Increased strength MMS > IFA Increased blood volume IFA > MMS Improved health of mother and baby ↔	High level of trust in doctors and public health system, free healthcare; fast delivery	Skipped supplement if no food available, especially in the MMS group. Rumors across all groups: fetus will grow too big, painful delivery and complications; in IFA only, some said that the supplement causes hemorrhage during delivery	Midwife at enrolment and health assistants for weekly home visits	Husbands
Aguayo [22]; Mali	↔	97%—easy to remember to take the supplement daily; encouraged by family and community members	Not measured	Home delivery—health worker from the nearest health facility	Community and husbands
Ba [26]; Mali	MMS > IFA; better health and nutrition, “strengthened” blood and health of the child, increased maternal appetite and weight gain, and healthier babies	Counseling materials and visual aids; calendar/tracking chart; family member influence (e.g., reminders); ‘Djigui’ [MMS] brand name and packaging adaptation for local culture	Concerns about the effect of MMS on the baby’s birthweight (“larger babies”); difficulty in opening bottles resulted in skipping doses; confusion if MMS is a nutrient, drug, or medication, and concerns associated with taking a drug during pregnancy; IFA not free of cost as SOC; suspicion regarding free provision of supplements; forgetting to take the supplement	ANC	Husbands
Sauer [29]; Cambodia	MMS > IFA; Increased energy, improved sleep; felt healthier; felt happier; liked the MMS packaging	Less of a burden to take MMS than IFA (99% vs. 94%, respectively)	Bad taste; bad smell; willingness to purchase the supplement	Healthcare workers at ANC	Not measured
Abebe [27]; Ethiopia *	50% said health benefits can make it easier for individuals to use MMS; 57% said it is essential to take MMS	Product name and packaging should be adapted for the local context; 21% said that MMS availability can improve acceptability; family members reminded them to take IFA	IFA: skipped supplement if no food; fear of side effects (30%); bad taste (21%); forgetfulness (15%); lack of awareness and support from family members; not free of cost	ANC (only IFA was distributed)	Not measured
Horino [28]; Jordan (UNRWA) Conference abstract	MMS > IFA: felt healthier and more energetic; better appetite	MMS > IFA: more acceptable among clinicians and pregnant individuals; fewer side effects; ease of use; similar cost for IFA and MMS	MMS > IFA; Less pleasant smell/taste;	ANC	Not measured

* The study tested IFA utilization, whereas for MMS their willingness to pay for MMS was assessed. ↔ no difference between groups; Abbreviations: ANC = Antenatal Care, IFA = Iron and Folic Acid Supplementation, MMS = Multiple Micronutrient Supplementation, SOC = Standard of Care.

## Data Availability

No new data were created or analyzed in this study. Data sharing is not applicable to this article.

## Supplementary Materials

The following supporting information can be downloaded at: https://www.mdpi.com/article/10.3390/nu17182994/s1, Figure S1: PRISMA flowchart of identified and included articles; Table S1: Search strategy employed on Ovid Medline; Supplementary Case Study S1: Assessing acceptance and adherence to UNIMMAP MMS among pregnant women in Philippines: Findings from a 2022–2023 evaluation; Supplementary Case Study S2: Advancing maternal nutrition: evaluating multiple micronutrient supplement acceptability in Sierra Leone’s ANC services; Supplementary Case Study S3: Study report: evaluation of the impact of community distribution of multiple micronutrients (MMN) in pregnant women from the Bla health district in 2023.

## Abbreviations

The following abbreviations are used in this manuscript:

ANC	Antenatal care
FA	Folic acid supplementation
IFA	Iron and folic acid supplementation
MMS	Multiple micronutrient supplementation
N/A	Not applicable
LMICs	Low- and middle-income countries
LNS	Lipid-based nutrient supplement
RCTs	Randomized controlled trials
UNIMMAP	United Nations International Multiple Micronutrient Antenatal Preparation
UNRWA	United Nations Relief and Works Agency for Palestine Refugees in the Near East
WHO	World Health Organization
