# Supplementary material for: Acceptability of Antenatal Multiple Micronutrient Supplementation (MMS) Compared to Iron and Folic Acid (IFA) Supplementation in Pregnant Individuals: A Narrative Review
Source: Nutrients. 2025 Sep 18;17(18):2994. doi: 10.3390/nu17182994 (PMC12472735; doi:10.3390/nu17182994)
Supplement: Supplementary file 1 [file nutrients-17-02994-s001.zip › nutrients-3855844-supplementary.pdf]

Table S1. Search strategy employed on Ovid Medline

1	exp Pregnancy/
2	(pregnan* or maternal or prenatal or antenatal).mp. [mp=title, abstract, heading word, drug trade name, original title, device manufacturer, drug manufacturer, device trade name, keyword heading word, floating subheading word, candidate term word]
3	1 or 2
4	((micronutrient* or multivitamin* or prenatal) adj3 supplement*).mp. [mp=title, abstract, heading word, drug trade name, original title, device manufacturer, drug manufacturer, device trade name, keyword heading word, floating subheading word, candidate term word]
5	multiple* micronutrient*.mp. [mp=title, abstract, heading word, drug trade name, original title, device manufacturer, drug manufacturer, device trade name, keyword heading word, floating subheading word, candidate term word]
6	4 or 5
7	acceptability.mp. [mp=title, abstract, heading word, drug trade name, original title, device manufacturer, drug manufacturer, device trade name, keyword heading word, floating subheading word, candidate term word]
8	(adherence or compliance or consumption or uptake).mp. [mp=title, abstract, heading word, drug trade name, original title, device manufacturer, drug manufacturer, device trade name, keyword heading word, floating subheading word, candidate term word]
9	7 or 8
10	3 and 6 and 9
11	(rat* or mouse or mice).mp. [mp=title, abstract, heading word, drug trade name, original title, device manufacturer, drug manufacturer, device trade name, keyword heading word, floating subheading word, candidate term word]
12	10 not 11

Figure S1. PRISMA flowchart of identified and included articles.

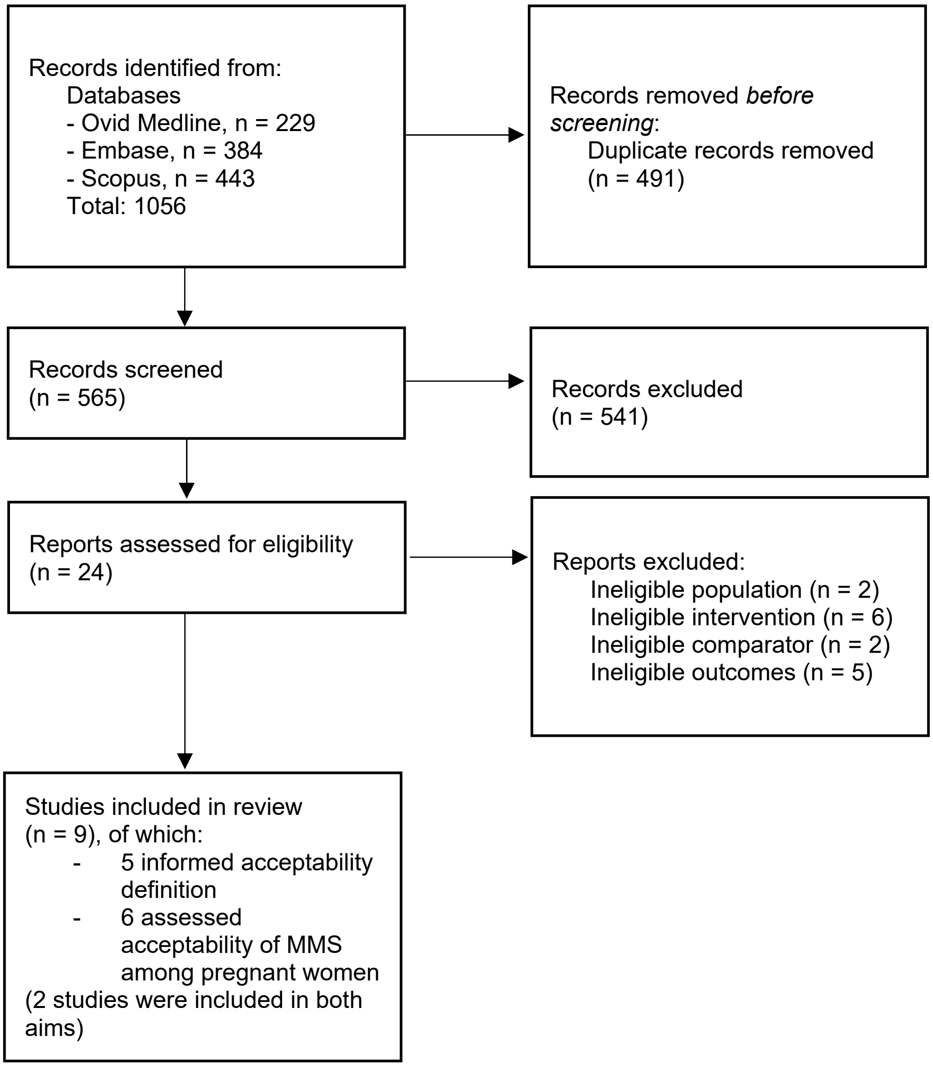

<u>Supplementary Case Study 1:</u> assessing acceptance and adherence to unimmap mms among pregnant women in Philippines: findings from a 2022-2023 evaluation

Dr. Luz Escubil, Aishwarya Panicker, and Dr. Ashutosh Mishra

Vitamin Angel Alliance

Efforts have been underway to provide United Nations International Multiple Micronutrient Antenatal Preparation (UNIMMAP MMS; henceforth referred to as MMS) to underserved pregnant women in Philippines since 2017. Initially, the program was through non-government organizations (NGOs) and later included local government units (LGUs). The Department of Health issued interim Nutritional Guidelines (DOH Department Memorandum 2020-0092) and drafted the Manual of Operations for micronutrient supplementation, recommending prescribing MMS to nutritionally at-risk pregnant women. In addition, recent high visibility activities like Buntis Congress emphasized the importance of MMS for healthy pregnancy and better birth outcomes.

A process evaluation from 2022 to 2023 documented the acceptance of MMS among pregnant women. Data collected were based on in-depth Interview (n=9) and focus group discussions (n=85). While MMS distribution was integrated into the maternal and child Health (MCH) program of LGUs, NGO partners included MMS in their MCH, Reproductive and Adolescent Health, Nutrition in Emergency, and Water, Sanitation, and Hygiene programs.

Acceptability of UNIMMAP MMS was examined in 89 pregnant women who were asked to record their MMS intake for 90 days. Majority liked the taste (82.8%) and color (71%) of the MMS tablet, along with its texture, smell, and size. Positive ratings were also given for preparation (85.7%), packaging (95.7%), and number of doses per bottle (90%). However, only a fourth consumed at least 90 tablets, with 73.0% reporting discontinuation or skipping MMS. Forgetfulness (54.4%) was a major reason, with some leaving their MMS bottles at home (13%) when traveling, and others (9%) stopping due to side effects.

Based on qualitative interviews with pregnant women, 5 of the 7 who used a pill dispenser (provided within the study) found it convenient and effective as a reminder. Pregnant women also reported that the provision of a tracking calendar (distributed as part of an informational MMS brochure) was a helpful reminder. The calendar also assisted in counselling and monitoring efforts by health professionals who were reported by pregnant women as a trusted source of information and reminders. These findings suggest that MMS delivery strategies by NGOs and government facilities in Philippines are acceptable, can be coordinated, and are complementary in nature.

Supplementary Case Study 2: Advancing maternal nutrition: evaluating multiple micronutrient supplement acceptability in Sierra Leone's ANC services

Advancing Maternal Nutrition: Evaluating Multiple Micronutrient Supplement Acceptability in Sierra Leone's ANC Services

Djeinam Toure[1]; Umu Jalloh[2]; Francis Moses[3]; Aminata Shamit Koroma[4]; Sugandh Juneja[2]; Mohamed Bangura[2]; Mansour Ndiath[5]; Garn Kristine[6]
[1]Africa region, Helen Keller Intl, Dakar, Senegal; [2]Sierra Leone, Helen Keller International, Freetown, Sierra Leone; [3]Reproductive Health and Family planning, Ministry of Health and Sanitation, Sierra Leone, Freetown, Sierra Leone; [4]Director of Food and Nutrition, Ministry of Health and Sanitation, Sierra Leone, Freetown, Sierra Leone; [5]MER, Helen Keller INTL - Regional & Senegal, Dakar, Senegal; [6]Helen Keller Intl, Helen Keller INTL - Regional & Senegal, Dakar, Senegal

Number: **ICNAB-01918**
Speaker: **Umu Jalloh**
Mode d'étude : **Abstract**
Thème : **5. Public health and nutrition throughout life cycle**
Keywords list (min. 1, max. 5) :

- Acceptability
- MMS
- Prenatal supplementation
- Sierra Leone

Type of presentation : **Oral presentation**
Conflicts of interest : **No**

- [x] Ethical Charter
- [x] Data Protection
- [x] Engagement
- [x] Recording acceptance

Updated on: **Friday, December 13, 2024 4:58 PM**

Background and objectives
There is significant evidence of the benefit of multiple micronutrient supplements (MMS) during pregnancy over iron folic acid (IFA) on maternal and infant health and nutrition. In view of this, the Ministry of Health (MoH) of Sierra Leone decided to switch from IFA to MMS in 2023 with the support of its partners Helen Keller International and CARE International. The MMS is distributed in bottles containing 180 tablets to address the problem of inconsistent supplementation among pregnant women, but the impact this may have on ANC attendance and the general acceptability of the product in Sierra Leone is unclear. To address these implementation questions, a formative study was carried out in three districts where Helen Keller Intl supported the government to transition from IFA.

Methods
A survey was conducted with 533 women in Kenema, Bombala, and Western Area districts in Sierra Leone. Women were enrolled at ANC and administered a survey questionnaire assessing their perceptions of MMS organoleptic, packaging, ease of use, social and time resources for supplement use and perceived impact on health. Women were also asked about how they valued ANC and their trust of ANC service providers.

Results
The study has four key findings: first, most women who attended ANC reported receiving MMS (88%) and counseling for MMS. Second, women value ANC beyond MMS receipt and did not perceive that a 180-pill ration would limit ANC attendance. This was evidenced by the high trust and value of the counseling offered at ANC. Third, women found the MMS supplement to be acceptable. Women liked the pill size and color, found packaging attractive, and found the product easy to use. Taste, smell and ease of swallow were slightly less accepted. Finally, social support and education can be strengthened. A quarter of women did not feel that their family supported them to take MMS. Though women generally were aware of MMS health benefits to themselves and their babies, some women were unaware of the need to continue taking the supplement even if their diet was adequate and others noted double dosing in case of a missed dose.

Conclusions
The study found MMS in a 180-pill bottle to be acceptable among women who attend ANC in Sierra Leone. Women trust and value their ANC providers and experience, beyond the receipt of supplements. Future efforts should proceed with the administration of the 180 pill bottle of MMS and invest in strategies to improve knowledge and social dynamics that constrain supplementation.

<u>Supplementary Case Study 3</u> *(translated from French to English with AI)*: **Study Report: evaluation of the impact of community distribution of multiple micronutrients (MMN) in pregnant women from the Bla health district in 2023**

Ministry of Health and Social Development, National Institute of Public Health, Mali

Investigators: Dr. Bakary Diarra, Prof Fatou Diawara, Dr. Djeneba Coulibaly, Yaya Togo, Ibrahim Terera, Abdoulaye S. Dabo, Mme Fanta Sacko

Executive Summary

The most common micronutrient deficiencies are iron, vitamin A, and iodine deficiencies, followed by zinc, folic acid (vitamin B9), vitamin B12, other B vitamins, vitamin C, vitamin D, calcium, selenium, and fluoride. These deficiencies during pregnancy are associated with an increased risk of maternal mortality, perinatal mortality, and low birth weight in newborns. Mali is no exception to this observation, as evidenced by various surveys.

Given the high prevalence of wasting among newborns and young infants, interventions targeting pregnant women play a role in its prevention, particularly iron and folic acid supplementation, which has clear benefits in preventing anemia during pregnancy. However, adequate intake of other vitamins and minerals among pregnant women, which are associated with adverse pregnancy outcomes, remains lacking.

The supplementation of multiple micronutrients (MMN) has been recommended by WHO and UNICEF as a more promising prenatal intervention. Considering this, replacing iron and folic acid supplementation with MMN supplementation within prenatal care for pregnant women in low- and middle-income countries is gaining consensus among leading nutrition experts.

Mali, like other countries, seeks to address micronutrient, vitamin, and mineral deficiencies to contribute to reducing maternal, neonatal, and infant mortality. To this end, it is exploring the potential impact of MMN supplementation on prenatal care and identifying the best strategies to reach the largest number of pregnant women.

In this context, the Ministry of Health and its partners have decided to introduce MMN supplementation in 15 health areas of the Bla Health District. This study, titled *"Evaluation of the Impact of Community Distribution of Multiple Micronutrients (MMN) Among Pregnant Women in the Bla Health District in 2023"*, aims to assess the mechanisms and effectiveness of platforms for the introduction of MMN in Mali. The goal is to provide reliable information to help authorities decide whether to adopt the strategy of MMN distribution in health areas based on the results from Bla.

The adopted methodology involved **a two-phase evaluation**. The first phase assessed the baseline status in terms of service utilization and prenatal care data and their analysis. The second phase reviewed the situation during MMN distribution. This mixed-methods (qualitative and quantitative) study included two groups: the first group described the situation of prenatal care coverage and the distribution of supplies in 7 CSComs (health centers), while the second group covered 8 health areas where similar data were evaluated to study variations. In this second group, distribution took place both at fixed centers and within the community.

Data collection under supervision took place from June 20 to 26, 2023, for the first phase, and from December 6 to 11, 2023, for the second phase. The targeted groups included pregnant women and healthcare workers, with information sources being activity reports and routine data from DISH2.

The study revealed that all sites, study populations, and information sources were fully covered. This included 15 healthcare facilities and their respective areas, 4 ASC (Community Health Worker) sites, 3 rural maternity centers per health area, 99 healthcare workers during the first phase and 95 in the second phase, and 624 pregnant women in the first phase and 626 in the second phase. All professional categories were represented among the healthcare workers.

Healthcare Workers' Perspectives on Iron and Folic Acid (FAF) vs. Multiple Micronutrients (MMN):
For both phases, healthcare workers surveyed included 44 from Group 1 and 108 from Group 2. Their average age was 39 years, with the youngest being 23 and the oldest 65. They had typically been in their roles for 1 to 4 years and included doctors, medical assistants, TSS (senior health technicians), TS (health technicians), midwives, pharmacy managers, and social workers.

- **MMN storage** was deemed easier by 88.5% of workers compared to 78.8% for FAF.
- **MMN transport** was also considered more manageable by 89.5% of workers compared to 71.7% for FAF.
- **Acceptance of MMN** was rated higher by women, with 97.9% favoring MMN compared to 67.7% for FAF.
  After the MMN distribution, all 95 healthcare workers surveyed in the second phase preferred MMN over FAF.

Preferred Distribution Strategies:
Among the workers, 40% recommended a community distribution strategy coupled with distribution at CSComs (Group 2 scenario), while 24.2% favored distribution exclusively at health centers (Group 1 scenario).

Pregnant Women's Perspectives:
Out of 1,250 women interviewed across both phases, 624 participated in the baseline study and 626 in the final study. Of these, 667 were from Group 1 areas.

- The average age of participants was 27, with the most represented age group being 20-24 years (30.08%), and ages ranging from 14 to 46.
- The majority of women (99.2%) were married, with 67.84% primarily engaged as homemakers. Most were uneducated, with 62% and 61.3% respectively in the first and second phases.
- Around 80.32% of the women had a daily income of less than $2 USD, classifying them as poor.
- Only 40.32% had decision-making autonomy regarding prenatal consultations (PNC), with the decision largely resting with their husbands (91.82%) or, to a lesser extent, their in-laws (5.49%).

Key Prenatal and Supplementation Statistics:

- Of the 1,250 women, 27.76% had attended at least one PNC session during their current pregnancy, while only 12.48% had attended at least four sessions. Meanwhile, 24% had not yet attended any PNC sessions at the time of the survey.
- 69.68% received Td2 vaccinations, and 30.72% received an LLIN (long-lasting insecticidal net).
- In the first phase, 59.3% of the 624 women had received FAF/IFA. During the second phase, 95.5% received MMN.
- Regarding taste, 87.3% of women found MMN to be pleasant, compared to only 45.9% for FAF/IFA.

A small proportion of women found the taste of MMN unpleasant (10.4%), compared to a higher proportion for FAF, approximately one-third (33.4%). Regarding the presence of side effects, 25.2% of the 624 women in the baseline study reported experiencing them, compared to only 0.7% of the 598 women who consumed MMN in both study groups during the final phase.

For FAF, the most frequently reported side effects were nausea (80 occurrences), vomiting (98 occurrences), bloating (2 occurrences), and dark stools (1 occurrence). For MMN, nausea (3 occurrences) and vomiting (1 occurrence) were the only reported side effects.

In the second phase of the study, of the 598 women who received MMN, 464 (77.6%) had also previously used FAF. MMN as a supplement was overwhelmingly preferred (98%) over FAF. The acceptance of MMN by husbands was higher (99.3%) compared to FAF. MMN also received greater acceptance from in-laws (99.3%) compared to FAF (81.3%).

Among the 464 women who experienced both supplements in both study groups, 441 (95%) preferred MMN over FAF (5%). They justified their preference with reasons such as better taste, ease of consumption, absence of side effects, a higher vitamin content, the ability to promote good health, and increased appetite.

Analysis of Prenatal Care (PNC) Coverage and Related Services:
Data analysis on PNC coverage and related services was conducted using the DISH2 database, comparing the period before MMN distribution (baseline study) to the distribution period (final study).

- **For PNC1 coverage**, there was an average increase from 57% (before MMN distribution) to 77.6% (after MMN distribution), representing a 21% rise. This increase was more pronounced in Group 2 (26%) compared to Group 1 (14%).
    - This suggests that MMN distribution could improve PNC1 coverage in both study groups, with a more significant impact in Group 2.
- **For PNC4 coverage**, there was no change between the two study phases, remaining at 29%. While there was a slight increase in Group 2 (2%), there was a decrease in Group 1 (-1%).
    - This lack of progress may point to challenges such as delayed PNC1 attendance, insufficient quality of PNC services to retain women for follow-up visits, and seasonal factors like the rainy season, which limits advanced strategies and reduces health service utilization due to farm work and difficult access to some health centers.
    - Overall, MMN distribution did not negatively impact PNC4 coverage.

Related PNC Services:

- MMN was distributed more effectively (102% coverage) on average across both groups than FAF (94%), with the increase being more significant in Group 2.

The assessment of PNC (Prenatal Consultation) effectiveness between the two phases of the study showed a decline in the coverage of LLIN distribution (long-lasting insecticidal nets), IPT/SP (intermittent preventive treatment with sulfadoxine-pyrimethamine), FAF (iron and folic acid), and Td2 (tetanus-diphtheria vaccination). This highlights the need to evaluate the effectiveness of PNC services.

The analysis of PNC1 coverage data (increased by 14% in Group 1 and 26% in Group 2) indicates that MMN distribution positively influences the use of PNC1 services, regardless of the distribution strategy. This outcome could be attributed to the intense communication campaign conducted during the MMN distribution as part of the project.

Whether MMN distribution occurred exclusively at fixed centers (Group 1) or at both fixed centers and within the community (Group 2), the majority of pregnant women accessed PNC services during the MMN distribution phase (final study).

Cost Analysis:
To cover the expected 1,119,774 pregnancies in the reference year 2023, Mali would need to allocate:

- **2,418,711,840 FCFA** to purchase 40,311,864 blister packs of 10 FAF tablets.
- **2,777,039,520 FCFA** to purchase 2,239,548 boxes of 180 MMN tablets.

These amounts are for sourcing the supplements only. Other cost components were considered similar for both supplements. Based on sourcing prices, FAF costs **0.87 times less** than MMN. Transitioning from FAF to MMN would result in an additional annual expenditure of **358,327,680 FCFA** for Mali.

Advantages of MMN:
Beyond economic considerations, MMN offers the added benefit of providing pregnant women with essential vitamins beyond FAF, helping to prevent anemia in both mothers and their children during pregnancy and postpartum, while supporting better growth for children.

Key Findings and Recommendations:

- Community distribution achieved higher coverage compared to distribution exclusively at health centers.
- Community distribution did not negatively impact the utilization of PNC services.
- Study participants, both women and healthcare providers, preferred MMN over FAF due to its multivitamin composition, the near absence of side effects, its pleasant taste, ease of consumption, health benefits, appetite stimulation, as well as easier storage and transportation.

Recommendations:
We suggest that the Malian health authorities responsible for maternal and child health adopt MMN as the supplementation strategy during pregnancy and postpartum. However, it is essential to conduct further studies, particularly qualitative ones, to understand the underutilization of perinatal services and the late recourse to healthcare services. These efforts will help contribute to the reduction of maternal and infant morbidity and mortality.
